# Cerebral Motor Functional Connectivity at the Acute Stage: An Outcome Predictor of Ischemic Stroke

**DOI:** 10.1038/s41598-018-35192-y

**Published:** 2018-11-14

**Authors:** Nai-Fang Chi, Hsiao-Lun Ku, David Yen-Ting Chen, Ying-Chi Tseng, Chi-Jen Chen, Ying-Chin Lin, Yi-Chen Hsieh, Lung Chan, Hung-Yi Chiou, Chung Y. Hsu, Chaur-Jong Hu

**Affiliations:** 1 Graduate Institute of Clinical Medicine, College of Medicine, Taipei Medical University, Taipei, Taiwan; 2 Department of Neurology, School of Medicine, College of Medicine, Taipei Medical University, Taipei, Taiwan; 3 Department of Neurology, Stroke Center, Shuang Ho Hospital, Taipei Medical University, New Taipei, Taiwan; 4 Faculty of Medicine, National Yang-Ming University School of Medicine, Taipei, Taiwan; 5 Brain and Consciousness Research Center, Taipei Medical University, Taipei, Taiwan; 6 Department of Psychiatry, School of Medicine, College of Medicine, Taipei Medical University, Taipei, Taiwan; 7 Department of Radiology, School of Medicine, College of Medicine, Taipei Medical University, Taipei, Taiwan; 8 Department of Family Medicine, Shuang Ho Hospital, Taipei Medical University, New Taipei, Taiwan; 9 Department of Family Medicine, School of Medicine, College of Medicine, Taipei Medical University, Taipei, Taiwan; 10 The PhD Program of Neural Regenerative Medicine, College of Medical Science and Technology, Taipei Medical University, Taipei, Taiwan; 11 School of Public Health, College of Medicine, Taipei Medical University, Taipei, Taiwan; 12 Department of Neurology, China Medical University Hospital, Taichung, Taiwan; 13 Graduate Institute of Neural Regenerative Medicine, College of Medical Science and Technology, Taipei Medical University, Taipei, Taiwan

## Abstract

Sixty-seven patients with first acute ischemic stroke onset between 3 to 7 days and 25 age- and sex- matched controls were analyzed for the performance of a resting-state functional MRI to investigate whether the functional connectivity (FC) of the motor network in acute ischemic stroke is independently associated with functional outcomes. The FC of cortical motor network and default mode network was analyzed. The FC was compared between controls, patients with favorable outcomes (modified Rankin Scale, mRS ≤1), and patients with unfavorable outcomes (mRS ≥2) at 3 months. Of the 67 patients, 23 (34%) exhibited unfavorable outcomes. In multivariate analysis, the FC between ipsilesional primary motor cortex (M1) and contralesional dorsal premotor area (PMd) ≤0.63, were independently associated with unfavorable outcomes (odds ratio = 6.32, *P* = 0.032), whereas the FC of default mode network was not different between groups. The interhemispheric FC of the motor network is an independent predictor of functional outcomes in patients with acute ischemic stroke.

## Introduction

Ischemic stroke is a leading cause of disability in developed countries. More than half patients with ischemic stroke can not go back to work despite months of aggressive treatment and rehabilitation^1^. Age, stroke severity, lesion volume, or lesion location are important factors influencing functional outcome; but none of them could be intervened effectively, and mild stroke severity does not guarantee a favorable outcome^2^. Several factors, such as uncontrolled hypertension, hyperglycemia, and inflammation, have been proposed to predict unfavorable functional outcomes^3–5^. However, these factors are also associated with stroke severity, and they may account for the consequences more than the causes of the unfavorable outcomes^3,6^. Most clinical trials targeting these factors have reported controversial results^7–9^. Therefore, it is crucial to explore novel and independent prognostic biomarkers for developing recovery treatment strategies.

Resting-state functional magnetic resonance imaging (fMRI) is a valid research tool for stroke^10^. With this technique, the functional connectivity (FC) represents the synchrony of intrinsic blood oxygen level-dependent (BOLD) signal fluctuations among different brain regions. A high FC means a good connection of neuronal activities between different brain regions. In patients with ischemic stroke, the FC of the motor network is impaired within hours after stroke onset^11^, and it changes with motor deficit improvements during longitudinal observations^12–14^. Therefore, the FC of the motor network is a potential imaging biomarker of stroke recovery. The limitations of fMRI in current stroke research are the lack of study in acute disease stage: Most previous studies regarding fMRI in stroke enrolled patients at 2 weeks or later after stroke onset^11–23^. Besides, previous studies had relatively small subject number in each study, and it is difficult to eliminate the influence of confounding factors of stroke outcome. Another issue is the potential hemodynamic lag in the hemisphere with ischemic lesions, which may affect the measurement of FC^24,25^. Some approaches such as cross-correlation are applied in detecting hemodynamic lags in the BOLD signal, and strategies to correct the influence of hemodynamic lags in fMRI are under investigation^26,27^.

We hypothesized that the FC of the motor network in patients with acute ischemic stroke is worse than health population, and the FC at acute stage of stroke is predictive of the functional outcomes.

## Methods

### Participants

This study was approved by the Institutional Review Board of Taipei Medical University. The data of this study are available from the corresponding author upon request. All methods were performed in accordance with relevant guidelines and regulations. From Aug 2015 to March 2017, patients with first acute unilateral ischemic stroke confirmed with neuroimaging and with a premorbid mRS score of 0 were consecutively screened when they were admitted to Taipei Medical University Shuang Ho Hospital within 7 days after stroke onset. Each patient was cared for according to standard clinical guidelines^28^.

MRI with T1- and T2-weighted images, T2 fluid-attenuated inversion recovery (T2 FLAIR) images, diffusion-weighted imaging (DWI), apparent diffusion coefficient maps, and time-of-flight magnetic resonance angiograms (MRA), carotid Doppler ultrasonography, and electrocardiogram of the patients were routinely evaluated. The exclusion criteria included: (1) Acute cortical infarction in the frontal or parietal lobe detected in DWI, which would overlap with the regions of interest (ROIs) of fMRI in this study (2) More than 50% stenosis of carotid, vertebral, or cerebral arteries (3) Pure sensory impairment at admission, which would usually have excellent functional outcome. Eighty-five patients were enrolled in this study, and 25 age- and sex-matched healthy volunteers were enrolled as controls. Written informed consent was obtained from all participants or their legal guardians. The etiologic subtype of stroke was classified according to the Trial of Org 10172 in Acute Stroke Treatment (TOAST) study^29^. An experienced neurologist (Chi) interpreted the lesion volume and location in DWI manually and rated pre-existing white matter hyperintensities in T2 FLAIR by using the Fazekas scale^30^. Resting-state fMRI was conducted within 7 days (range: 3 to 7 days) after stroke for the patients and once for the controls. The NIHSS was administered on the same day as the resting-state fMRI, and the mRS was evaluated at 3 months (range: 80 to 105 days). The patients with a mRS score of ≤1 at 3 months were defined as having favorable outcome, whereas those with a mRS score of ≥2 at 3 months were defined as having unfavorable outcome.

### Resting-State Functional Magnetic Resonance Imaging

The images were acquired on a GE Discovery MR750 3.0 T scanner (General Electric Healthcare, USA) with a gradient echo planar imaging sequence by employing the following imaging parameters: flip angle, 90 degrees; repetition time (TR) = 2000 ms; echo time = 30 ms; field of view, 230 mm; matrix, 64 × 64 (in-plane resolution = 3.6 × 3.6 mm^2^); slice thickness, 3.0 mm; spacing, 1.0 mm; and scanning time, 360 seconds (180 imaging frames). Each participant had 1 run of imaging acquisition. The imaging acquisition was interleaved and from bottom to top. The subjects were asked to open their eyes and look at a fixed point in front of them during the scan.

### Functional Connectivity Analysis

#### Preprocessing

Preprocessing was performed using Statistical Parametric Mapping (SPM12; http://www.fil.ion.ucl.ac.uk/spm/software/spm12) and Data Processing Assistant for Resting-State fMRI V4.3 (DPARSF; http://rfmri.org)^31^. All scans were slice timing corrected, head movement corrected, and normalized to the eco-planar imaging template provided by SPM12. Spatial smoothing was performed using a 6-mm full-width half-maximum Gaussian kernel.

After spatial smoothing, potential hemodynamic lags in fMRI were detected by using Regressor Interpolation at Progressive Time Delays (RIPTiDe; https://github.com/bbfrederick/rapidtide)^32,33^. It uses cross-correlation to find the time course of endogenous low frequency oscillation (LFO, 0.009 to 0.15 Hz) in the mean BOLD signal, which is thought to be the signal of cerebral blood flow, and then it determines the strength and peak time lag of LFO at each voxel. We used RIPTiDe to produce a lag time map of whole brain in each subject. We calculated the average hemodynamic lag of the ROIs at each side of brain, and patients with a lag time difference more than 2 seconds (1 TR) between bilateral sides were excluded in this step (n = 5). In addition, RIPTiDe provides a function named “dynamic Global Signal Regression (dGSR)”^34^, which generate voxel-specific regressors to filter the signal of cerebral blood flow out of each voxel, therefore it can eliminate the influence of hemodynamic lag when calculating FC. In this study, although we had excluded the patients with substantial lateralized hemodynamic lags, certain hemodynamic lags at some ROIs might still exist. Therefore, in this study we processed the data and presented both the results with and without dGSR.

After preprocessing with and without dGSR, the following procedures were performed using the Resting-State fMRI Data Analysis Toolkit V1.8 (http://www.restfmri.net)^35^ and DPARSF V4.3. Nuisance signals were eliminated through regression of the following variables: (1) the 6 head movement parameters computed through rigid body translation and rotation during preprocessing, (2) the mean signal of cerebrospinal fluid, and (3) the mean signal within the white matter. After nuisance signals regression, low-frequency time course representing neuronal activities were extracted using a band-pass filter (0.01 to 0.08 Hz).

The framewise displacement (FD) was computed from the 6 head movement parameters. Image frames with FD > 0.5 mm were marked as unacceptable head motion, and the frames 1 back and 2 forward from the marked frames were excluded from FC analysis (image scrubbing)^36^. Patients with less than 150 image frames (5 minutes) after image scrubbing were excluded in this step (n = 13). Total 67 patients (44 patients with favorable outcomes, 23 patients with unfavorable outcomes) and 25 controls were enrolled for final analysis.

#### Selection of Regions of Interest (ROIs)

We selected the 5 cortical ROIs of the motor network reported by Wang *et al*.^12^, including primary motor cortex (M1), supplementary motor area (SMA), postcentral gyrus (PCG), ventrolateral premotor cortex (PMv), and dorsolateral premotor cortex (PMd). In addition, we selected the default mode network (DMN) to compare with the performance of motor network. The activity of DMN is prominent in resting state and DMN does not involve much about motor or language function, therefore it is suitable as the control network in this study. We selected 5 ROIs of DMN, including posterior cingulate cortex (PCC), anterior medial prefrontal cortex (amPFC), inferior parietal lobule (IPL), retrosplenial cortex (Rsp), and anterior hippocampus (AnHip). The ROI coordinates were based on the Montreal Neurological Institute system, and the ROIs represented 6-mm-radius spheres. All ROI coordinates used in this study are provided in the Supplementary Material. The ipsilesional ROIs were expressed as “iROI” (e.g., iM1), and the contralesional ROIs were expressed as “cROI” (e.g., cM1).

#### Extraction of the Time Courses of ROIs and Construction of the Resting State Network

The time courses of each ROI were extracted. The Pearson correlation coefficient of the average time courses for each ROI pair was calculated as the FC, and Fisher’s z transformation was applied to convert the FC to normal distribution. The FC between all ROIs of the motor network were calculated for each subject. The FC between ROIs was expressed as “the FC of ROI/ROI” (e.g., FC of iM1/cM1). The FC matrices and anatomic distribution figures were produced by using BrainNet Viewer toolbox V1.53^37^.

### Statistical Analyses

**Figure 1:**
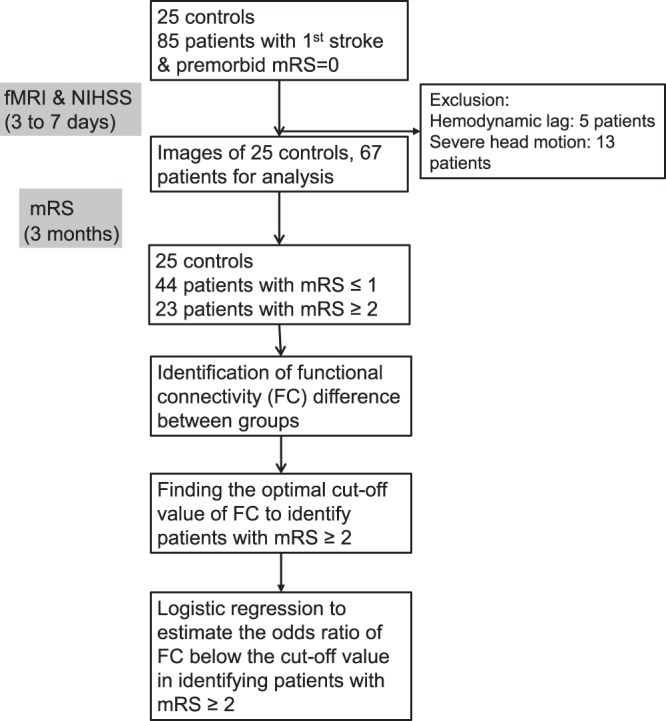
Flowchart of study protocol.

Data normality was tested by using the Shapiro-Wilk test. The demographic data between controls, patients with favorable outcomes, and patients with unfavorable outcomes were compared by using analysis of variance (ANOVA), the Kruskal-Wallis test, or Chi-square test and post hoc analysis. The FC between groups were compared by ANOVA, the false discovery rate (FDR) corrected *P* < 0.05 was considered statistically significant^38^. The Tukey-Kramer test was applied as the post hoc analysis when *P* value of ANOVA was considered statistically significant. The FC which was significantly different between the patients with favorable and unfavorable functional outcomes were chosen for further analysis: The receiver operating characteristic (ROC) analysis was used for testing the sensitivity and specificity of the FC, as well as its best cut-off value in identifying patients with unfavorable functional outcomes. A univariate logistic regression analysis was conducted to estimate the odds ratio (OR) of unfavorable functional outcomes for the demographic variables and FC. Age, sex, and variables with *P* ≤ 0.10 in the univariate logistic regression analysis were included in a multivariate logistic regression model to adjust the OR of unfavorable functional outcomes. In order to ensure the independence between variables included in the multivariate logistic regression, the variables were put in a multiple linear regression model (dependent variable: mRS at 3 months) to check the variance inflation factor (VIF), and the variable with a VIF ≥ 10 will be excluded from multivariate logistic regression to avoid the problem of multicollinearity. Normally distributed data are expressed as mean ± standard deviation, non-normal data are expressed as the median value and interquartile range (IQR). *P* < 0.05 was considered statistically significant. Statistical data were analyzed using MedCalc Statistical Software V17 (MedCalc Software bvba, Ostend, Belgium). The flowchart of study protocol is presented in the Fig. 1.

## Results

**Figure 2:**
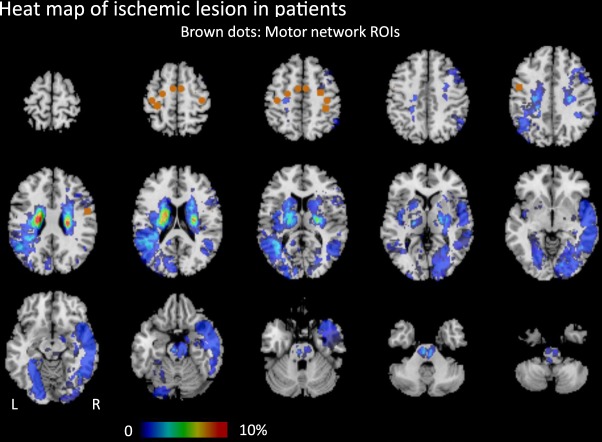
Heat map of ischemic lesion in patients.

**Figure 3:**
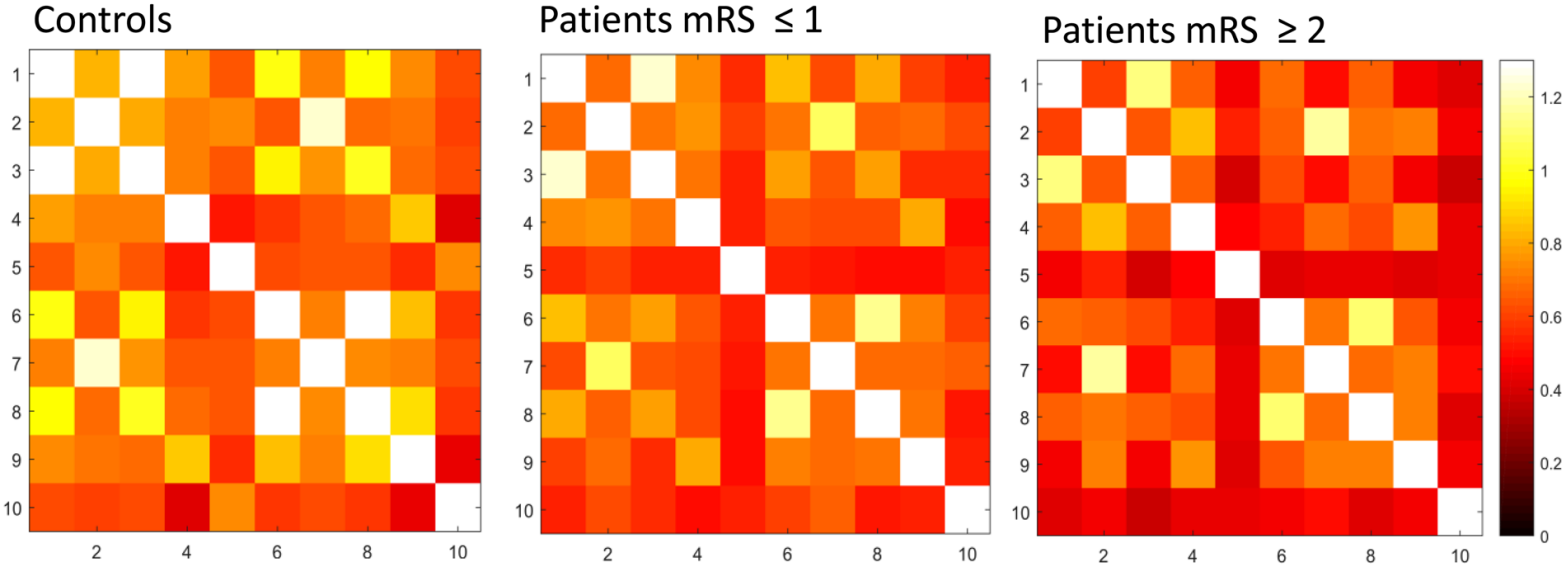
Motor network functional connectivity matrices (mean) ROIs: 1 = iM1, 2 = iSMA, 3 = iPCG, 4 = iPMd, 5 = iPMv 6 = cM1, 7 = cSMA, 8 = cPCG, 9 = cPMd, 10 = cPMv.

**Figure 4:**
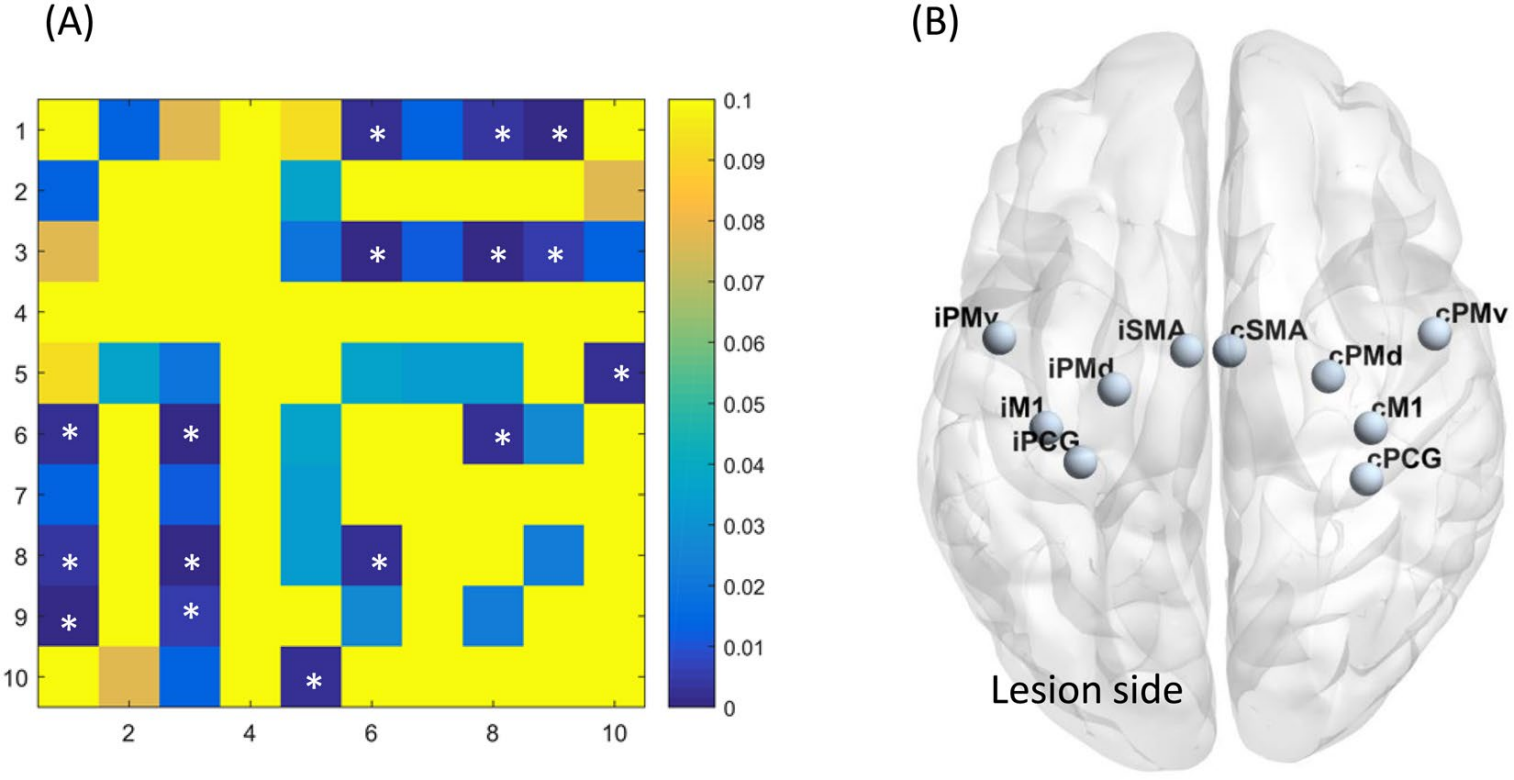
(**A**) The ANOVA P value matrices of the 3-group comparison in motor network functional connectivity ROIs: 1 = iM1, 2 = iSMA, 3 = iPCG, 4 = iPMd, 5 = iPMv 6 = cM1, 7 = cSMA, 8 = cPCG, 9 = cPMd, 10 = cPMv *FDR corrected P < 0.05 (**B**) Motor network ROIs.

**Figure 5:**
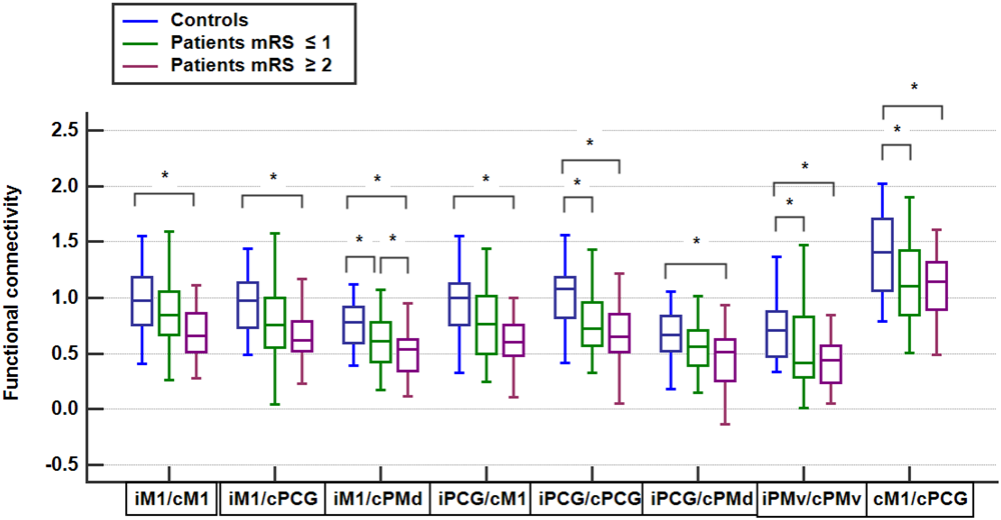
Functional connectivity comparison between groups *Significant difference in the post hoc test of ANOVA.

**Figure 6:**
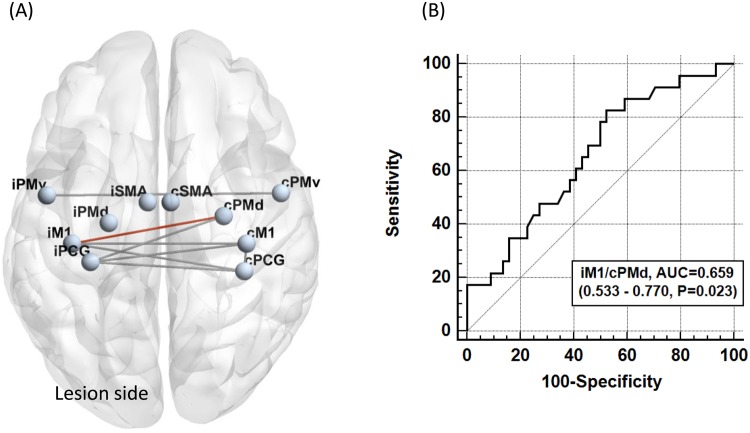
(**A**) Motor functional connectivity (FC) different between groups Gray lines: FC different between controls and patients Red line: FC different between patients with favorable and unfavorable outcomes (**B**) Receiver operating characteristics curve of iM1/cPMd in identifying patients with unfavorable outcome (mRS ≥ 2) The sensitivity = 82.6% and specificity = 47.7%, the optimal cut-off value = 0.63.

The clinical characteristics of the controls (n = 25), patients with favorable outcomes (n = 44), and patients with unfavorable outcomes (n = 23) are summarized in Table 1. No significant differences were observed in age and sex among the three groups. The proportion of patients with hypertension and hyperlipidemia was significantly higher than in controls. The proportion of having corticospinal tract lesion in DWI and the stroke etiologies were not different between patients with favorable and unfavorable outcomes. The most common stroke etiology was small vessel disease, followed by large artery atherosclerosis. Most patients had mild stroke severity (NIHSS: median = 3 in patients with favorable outcome, 4 in patients with unfavorable outcomes), small lesion volume in DWI (median = 0.60 cm^3^ in patients with favorable outcomes, 1.40 cm^3^ in patients with unfavorable outcomes), and mild pre-existing white matter hyperintensities (median score of periventricular and deep white matter Fazekas scale = 1 and 1 respectively).The spatial distribution of ischemic lesions in patients is presented in the Fig. 2. The FC matrices of motor network are presented in the Fig. 3, and the corresponding ANOVA *P* value matrices of the 3-group comparison are presented in the Fig. 4. The FC of iM1/cM1, iM1/cPCG, iM1/cPMd, iPCG/cM1, iPCG/cPCG, iPCG/cPMd, iPMv/cPMv, and cM1/cPCG were significantly different between the three groups. In the post hoc test of ANOVA (Fig. 5), all aforementioned FC were significantly lower in patients with unfavorable outcomes than in controls, and FC of iM1/cM1, iM1/cPCG, iPCG/cM1, and iPCG/cPMd were not different between patients with favorable outcomes and controls. Only the FC of iM1/cPMd was significantly lower in patients with unfavorable outcomes than in patients with favorable outcomes. The FC with significant differences between the controls and patients are presented in Fig. 6A.

**Table 1:** Clinical characteristics of the participants.

Characteristics	Controls (n = 25)	Patients with favorable outcomes (n = 44)	Patients with unfavorable outcomes (n = 23)	*P* value
Age, median (IQR)	57 (55–61)	61 (52–65)	59 (56–75)	0.247
Male Sex	16 (64%)	30 (68%)	12 (52%)	0.604
Hypertension	10 (40%)	29 (66%)^†^	21 (91%)^‡^	0.001*
Diabetes Mellitus	5 (20%)	13 (30%)	9 (39%)	0.347
Hyperlipidemia	8 (32%)	30 (68%)^†^	15 (65%)^†^	0.010*
Atrial fibrillation	0 (0%)	3 (7%)	1 (4%)	0.410
Lesion on the left side		22 (50%)	15 (65%)	0.143
NIHSS, median (IQR)		3 (1–4)	4 (3–9)	0.005*
mRS at 3 months, median (IQR)		1 (1-1)	2 (2–3)	<0.001*
DWI lesion at the corticospinal tract		25 (57%)	18 (78%)	0.206
DWI lesion volume in cm^3^, median (IQR)		0.60 (0.34–1.68)	1.40 (0.67–2.08)	0.124
Stroke etiology				0.858
Large artery atherosclerosis		12 (27%)	6 (26%)	
Small vessel disease		28 (64%)	16 (70%)	
Cardioembolism		3 (7%)	1 (4%)	
Other and undetermined etiologies		1 (2%)	0 (0%)	
White matter hyperintensities in T2 FLAIR				
Periventricular Fazekas scale, median (IQR)	0 (0–0)	1 (0–1)^†^	1 (0–2)^†^	0.014*
Deep white matter Fazekas scale, median (IQR)	0 (0–1)	1 (1–1)^†^	1 (0–2)^†^	<0.001*

*P < 0.05, ^†^different from controls, ‡different from both controls and patients with favorable outcomes in post hoc analysis.

IQR: interquartile range, mRS: modified Rankin Scale, NIHSS: National Institute of Health Stroke Scale.

ROC analysis showed that the area under curve (AUC) of the FC of iM1/cPMd was 0.659 [95% confidence interval (CI) = 0.533–0.770, *P* = 0.023] (Fig. 6B). The sensitivity and specificity of identifying the patients with unfavorable outcomes was 82.6% and 47.7% in the FC of iM1/cPMd, and the optimal cut-off value was 0.63. Therefore, the FC of iM1/cPMd ≤ 0.63 is a valid predictor of unfavorable functional outcomes in acute ischemic stroke.

**Figure 7:**
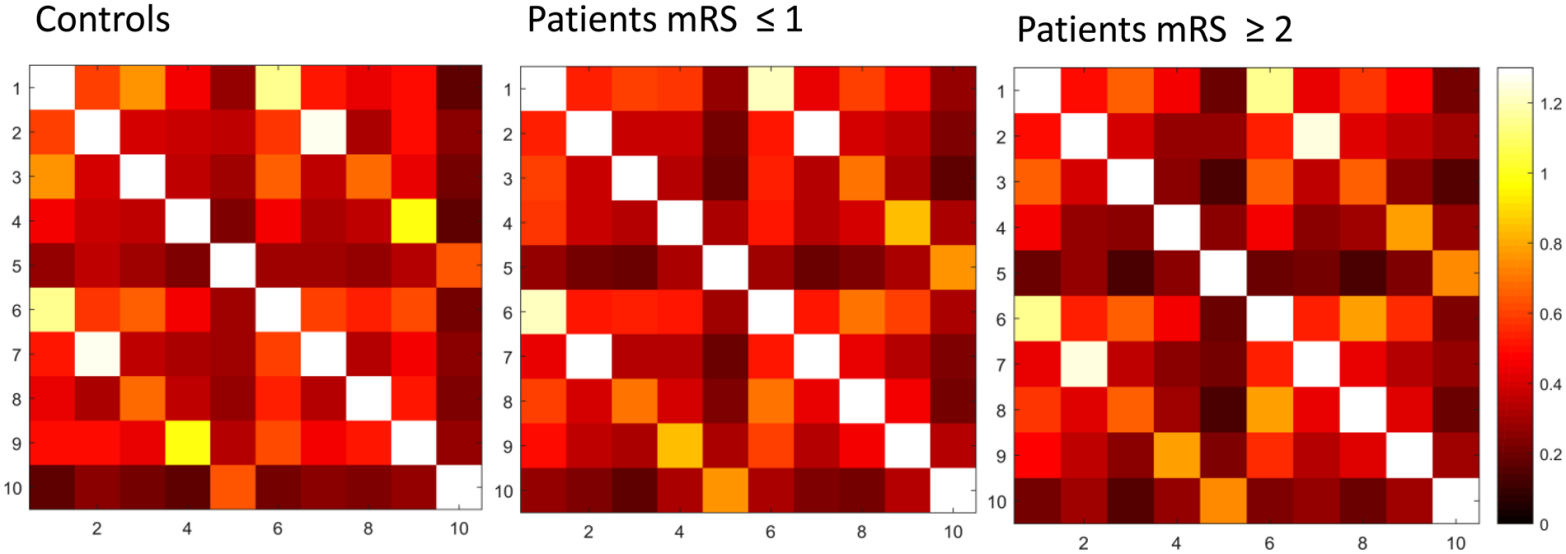
Default mode network functional connectivity matrices (mean) ROIs: 1 = iPCC, 2 = iamPFC, 3 = iIPL, 4 = iRsp, 5 = iAnHip 6 = cPCC, 7 = camPFC, 8 = cIPL, 9 = cRsp, 10 = cAnHip.

**Figure 8:**
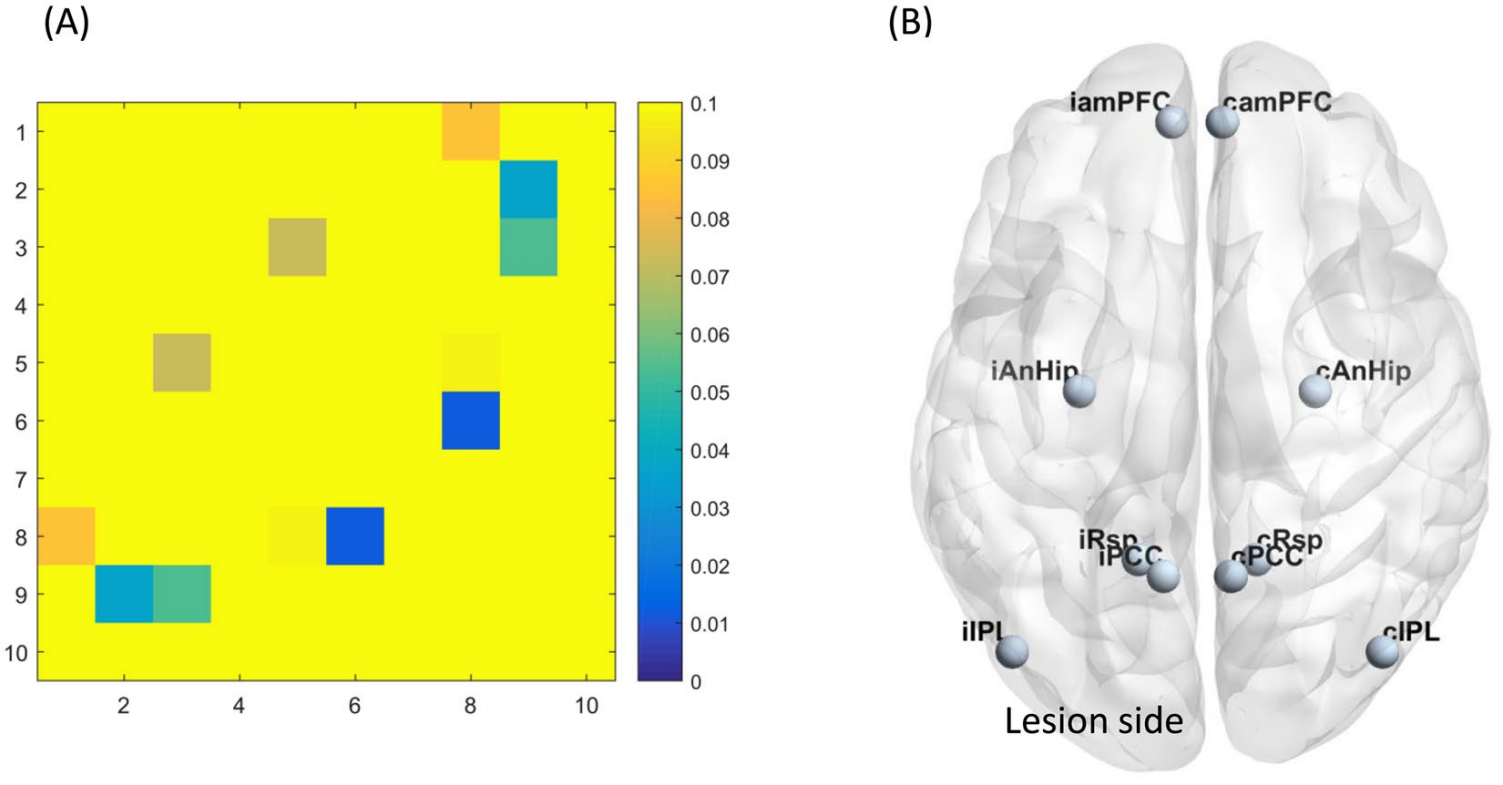
(**A**) The ANOVA P value matrices of the 3-group comparison in default mode network functional connectivity ROIs: 1 = iPCC, 2 = iamPFC, 3 = iIPL, 4 = iRsp, 5 = iAnHip 6 = cPCC, 7 = camPFC, 8 = cIPL, 9 = cRsp, 10 = cAnHip No significant difference existed after FDR correction (**B**) Defualt mode network ROIs.

The FC matrices of DMN are presented in the Fig. 7, and the corresponding ANOVA P value matrices of the 3-group comparison are presented in the Fig. 8. The FC of between any 2 ROIs in DMN are not significantly different between the three groups after FDR correction. Therefore, the FC of DMN is not predictive of functional outcome in acute ischemic stroke. In addition, it provide an evidence that the motor network FC difference between the three groups was not due to the presence of global physiological noises such as motions in a specific group.

**Figure 9:**
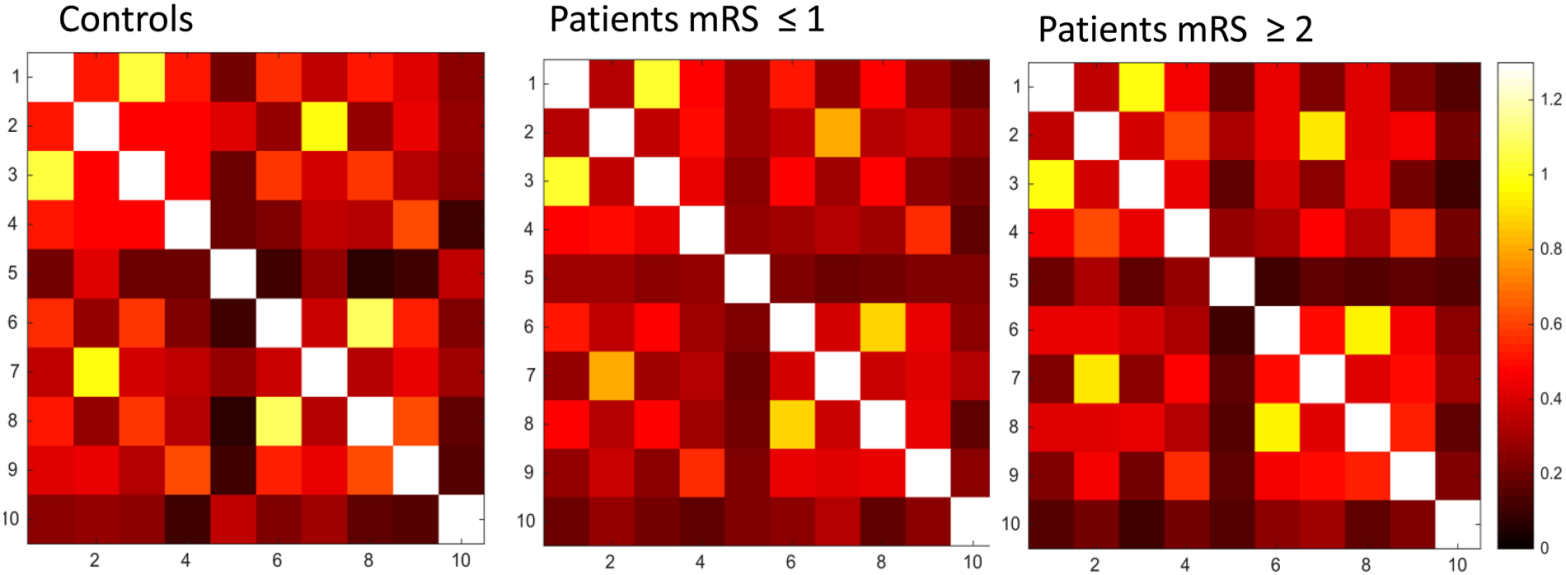
Motor network functional connectivity matrices (mean) by using dynamic Global Signal Regression ROIs: 1 = iM1, 2 = iSMA, 3 = iPCG, 4 = iPMd, 5 = iPMv = cM1, 7 = cSMA, 8 = cPCG, 9 = cPMd, 10 = cPMv.

**Figure 10:**
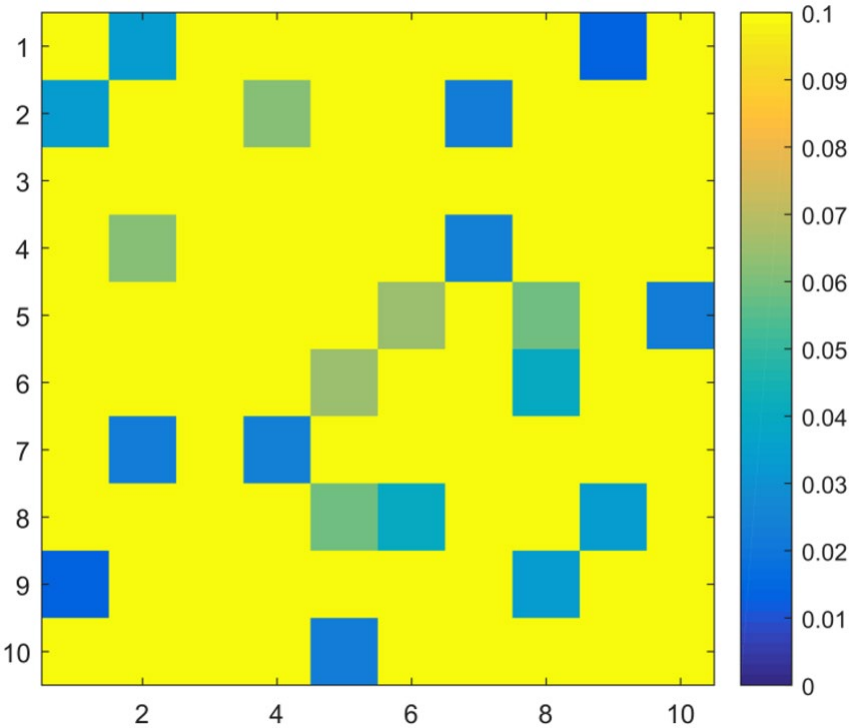
The ANOVA P value matrices of the 3-group comparison in motor network by using dynamic Global Signal Regression ROIs: 1 = iM1, 2 = iSMA, 3 = iPCG, 4 = iPMd, 5 = iPMv 6 = cM1, 7 = cSMA, 8 = cPCG, 9 = cPMd, 10 = cPMv No significant difference existed after FDR correction.

The FC matrices of motor network by using dGSR are presented in the Fig. 9, and the corresponding ANOVA *P* value matrices of the 3-group comparison are presented in the Fig. 10. The FC of between any 2 ROIs in motor network are not significantly different between the three groups after FDR correction.

Table 2 presents the results of univariate and multivariate logistic regression analyses of variables potentially predicting unfavorable functional outcomes. In univariate logistic regression analysis, hypertension, lesion at the corticospinal tract, NIHSS score, and the FC of iM1/cPMd ≤0.63 were significant predictors, whereas age, sex, diabetes, hyperlipidemia, atrial fibrillation, DWI lesion on the left side, DWI lesion volume, and Fazekas scale were not significant predictors. There was weak association between iM1/cPMd and age (correlation coefficient = −0.225, *P* = 0.031). In the multicollinearity test, age, sex, hypertension, lesion at the corticospinal tract, and NIHSS score had small VIF (<1.2), therefore, they were included in the multivariate logistic regression analysis to adjust the OR of the FC of iM1/cPMd ≤0.63. In the multivariate analysis, age and sex became significant predictors, whereas lesion at corticospinal tract became non-significant. The FC of iM1/cPMd ≤0.63 and NIHSS score remained significant predictors of unfavorable functional outcomes. In this multivariate logistic regression, the Nagelkerke R^2^ was 0.604. Therefore, the FC of iM1/cPMd is an independent outcome predictor among the FC in patients with acute ischemic stroke.

**Table 2:** Univariate and multivariate logistic regression analyses of clinical characteristics and functional connectivity to unfavorable neurological outcome (n = 67).

Characteristics	Unfavorable neurological outcome (mRS ≥ 2 at 3 months)			
	Crude OR (95% CI)	*p* value	Adjusted OR (95% CI)^†^	*p* value
Age	1.02 (0.98–1.08)	0.212	1.11 (1.02–1.20)	0.019*
Male sex	0.51 (0.18–1.43)	0.201	0.14 (0.02–0.79)	0.026*
Hypertension	5.43 (1.12–26.33)	0.036*	18.68 (1.62–215.13)	0.019*
Diabetes Mellitus	1.53 (0.53–4.42)	0.429		
Hyperlipidemia	0.88 (0.30–2.54)	0.806		
Atrial fibrillation	0.62 (0.06–6.33)	0.688		
DWI lesion on the left side	1.88 (0.66–5.31)	0.237		
DWI lesion at the corticospinal tract	3.58 (1.19–10.74)	0.023*	4.57 (0.77–27.12)	0.094
DWI lesion volume	1.02 (0.98–1.07)	0.331		
Periventricular Fazekas scale	1.33 (0.77–2.30)	0.289		
White matter Fazekas scale	1.35 (0.66–2.75)	0.406		
NIHSS	1.42 (1.14–1.78)	0.002*	1.74 (1.23–2.47)	0.002*
FC of iM1/cPMd ≤ 0.63	4.34 (1.27–14.84)	0.019*	6.32 (1.17–34.10)	0.032*

*P < 0.05, ^†^Adjusted for age, sex, hypertension, lesoin on corticospinal tract, NIHSS, and FC of iM1/cPMd ≤ 0.63.

## Discussion

Resting-state fMRI was proposed as a potential tool for predicting stroke outcomes^10^. The advantage of using resting-state fMRI instead of task-based fMRI in patients with stroke is that it can minimize the interindividual differences in the task performance and easily be integrated into routine clinical MRI. Previous studies have reported that FC improves with neurological functional recovery during longitudinal follow-up^12–15,17,39,40^, and FC is associated with neurological scores at certain observation time points^16,19,21,41,42^. Most previous studies regarding fMRI in stroke enrolled patients at 2 weeks or later after stroke onset with about 20 patients in each study^11–23^, so it was not possible to test the predictive power of FC by using multivariate analyses. This study analyzed 67 patients with acute ischemic stroke and mild severity, so it was able to find an optimal cut-off value of FC that predicts functional outcomes, and using multivariate analyses could clarify its association with other risk factors of unfavorable functional outcomes.

The disturbance in FC after mild to moderate ischemic stroke has been reported either within the ipsilesional hemisphere or between hemispheres^11–13,39^. Using resting-state fMRI, we determined that the interhemispheric FC of motor network in patients was lower than that in controls. However, only the FC of iM1/cPMd was significantly different between the patients with favorable and unfavorable functional outcomes (Figs 5 and 6). In studies using iM1 as the primary ROI, it was found that the interhemispheric FC increased and ipsilesional intrahemispheric FC decreased after rehabilitation^39^. In addition, decreased FC between bilateral motor cortices correlated with the neurological functions more favorably than that within ipsilesional motor cortices did^11,16,17^. Therefore, the interhemispheric FC might be more important than the ipsilesional intrahemispheric FC in the functional recovery of ischemic stroke. The PMd belongs to the Brodmann area 6, and it accounts for integrating the sensory information and movement targeting^43^. In a study of patients with stroke, contralesional PMd is more active compared with in healthy controls, which is thought to support the motor recovery^44^, and inhibitory repetitive transcranial magnetic stimulation (rTMS) of contralesional PMd worsen the recovered performance of paretic hand^45^. Therefore, it is reasonable that the FC of iM1/cPMd play a crucial role in the recovery of stroke.

In our study, the FC of iM1/cPMd at the acute stage of ischemic stroke independently predicted the functional outcome. Therefore, FC in fMRI appears to be a valid and timely imaging biomarker for acute ischemic stroke. Moreover, the FC is a potentially treatable target in stroke patients. By using neurostimulation techniques such as high frequency rTMS or anodal transcranial direct current stimulation (tDCS), the excitability of the motor cortices can be enhanced. However, the therapeutic effect of rTMS or tDCS in stroke remains controversial^46,47^. This may be explained by the lack of appropriate patient selection in past studies. The FC of motor cortices assessed by using resting state fMRI can help identifying the patients with high risk of unfavorable functional outcome, who may truly benefit from neurostimulation therapies.

Although the FC of iM1/cM1, iM1/cSMA, iM1/iPMd, and iM1/cPMd were reported to be correlated with patients’ neurological function in the chronic stage of stroke^17,39,44,48,49^. only the FC of iM1/cPMd was found to be significantly different between the patients with favorable and unfavorable functional outcomes in this study. An explanation is the difference of neurological rating scale between studies: most studies used the Fugl–Meyer Assessment (FMA) or only the motor domain of FMA, whereas we used NIHSS and mRS. The FMA is a more sophisticated rating scale than the mRS or NIHSS, therefore the FMA might be more sensitive in detecting the interindividual difference of neurological function. Nevertheless, the NIHSS and mRS are the most commonly used neurological rating scales in clinical studies of stroke, and the definition of mRS ≤ 1 as a favorable functional outcome has been commonly used. The FMA may be less amenable in clinical practice because it is more complex and requires a substantially longer administration time compared with the NIHSS or mRS^50^.

An interesting finding of this study is that after using dGSR, the differences of moto network FC between the three groups disappeared, although we had excluded the patients with substantial lateralized hemodynamic lags in advance. It is possible that the FC differences were driven by hemodynamic lags at some but not all ROIs of motor network, and dGSR eliminated the influence of hemodynamic lag to FC, therefore the FC abnormalities in patients were restored. In previous studies of FC in stroke, hemodynamic lags were managed by different approaches: 1. Excluding the subjects with lateralized hemodynamic lags^51^. 2. Shifting the time courses of ROIs with a hemodynamic lag to re-align with the global signal^27^, and 3. Using voxel-specific regressors to filter cerebral blood flow signal out of each voxel (dGSR)^33^. In this study, the motor network FC differences between groups existed after excluding the subjects with lateralized hemodynamic lags, but disappeared after using dGSR. Therefore, different managements of a hemodynamic lag result in different conclusions, and it is worth more investigations in the management of hemodynamic lag in fMRI.

In this study the DWI lesion volume was not a significant outcome predictor in the regression analysis. This result is not consistent with the common sense that large infarction volume is associated with poor outcome. The most probable reason of this result is that the lesion volume in our patients were consistently small, and some patients had large lesions at non-motor area such as posterior cerebral artery territory. This point of view is supported by the non-significant correlation between lesion volume and NIHSS score in our patients (correlation coefficient = 0.187, *P* = 0.129). Therefore, it is reasonable that the lesion volume is not predictive of outcome in our data.

This study has limitations. First, we excluded patients with frontal or parietal cortical infarction that could involve the ROIs in this study, as well as patients with significant vascular stenosis. The current fMRI analytic methods would raise concerns of inaccurately calculating FC in patients with lesions in ROIs or with significant vascular stenosis. Second, the mechanism underlying poor FC in patients with unfavorable outcomes compared with those with favorable outcomes remains unclear and could not be identified in the present study. Third, there is no validation test of the cut-off FC values in this study, which will need a new group of patients to validate our findings.

In conclusion, the cortical FC of the motor network is impaired in patients with acute ischemic stroke; in particular, the FC of iM1/cPMd at the acute stage is independently associated with functional outcomes. These findings require validation in another group of patients.

## Electronic supplementary material

Supplementary material

## Data Availability

The datasets generated and analysed during the current study are available from the corresponding author on reasonable request.
