# Supplementary material for: Cerebral Motor Functional Connectivity at the Acute Stage: An Outcome Predictor of Ischemic Stroke
Source: Sci Rep. 2018 Nov 14;8:16803. doi: 10.1038/s41598-018-35192-y (PMC6235876; doi:10.1038/s41598-018-35192-y)
Supplement: Supplementary file 1 — Supplementary material [file 41598_2018_35192_MOESM1_ESM.docx]

**Title**

Cerebral Motor Functional Connectivity at the Acute Stage: An Outcome Predictor of Ischemic Stroke

**Authors**

Nai-Fang Chi, MD;^1,2,3,4,5^ Hsiao-Lun Ku, MD;^5,6*^ David Yen-Ting Chen, MD;^5,7*^

Ying-Chi Tseng, MD;^5,7^ Chi-Jen Chen, MD;^5,7^ Ying-Chin Lin, MD;^8,9^, Yi-Chen Hsieh, PhD;^10^ Lung Chan, MD, PhD;^2,3^ Hung-Yi Chiou, PhD;^1,11^ Chung Y Hsu, MD, PhD;^12^ and Chaur-Jong Hu, MD ^1,2,3,13^

1 Graduate Institute of Clinical Medicine, College of Medicine, Taipei Medical University, Taipei, Taiwan

2 Department of Neurology, School of Medicine, College of Medicine, Taipei Medical University, Taipei, Taiwan

3 Department of Neurology, Stroke Center, Shuang Ho Hospital, Taipei Medical University, New Taipei, Taiwan

4 Faculty of Medicine, National Yang-Ming University School of Medicine, Taipei, Taiwan

5 Brain and Consciousness Research Center, Taipei Medical University, Taipei, Taiwan

6 Department of Psychiatry, School of Medicine, College of Medicine, Taipei Medical University, Taipei, Taiwan

7 Department of Radiology, School of Medicine, College of Medicine, Taipei Medical University, Taipei, Taiwan

8 Department of Family Medicine, Shuang Ho Hospital, Taipei Medical University, New Taipei, Taiwan

9 Department of Family Medicine, School of Medicine, College of Medicine, Taipei Medical University, Taipei, Taiwan.

10 The PhD Program of Neural Regenerative Medicine, College of Medical Science and Technology, Taipei Medical University, Taipei, Taiwan

11 School of Public Health, College of Medicine, Taipei Medical University, Taipei, Taiwan

12 Department of Neurology, China Medical University Hospital, Taichung, Taiwan

13 Graduate Institute of Neural Regenerative Medicine, College of Medical Science and Technology, Taipei Medical University, Taipei, Taiwan

* Drs Ku and Chen contributed to this study equally.

**Correspondence to:**

Chaur-Jong Hu MD

Graduate Institute of Clinical Medicine, College of Medicine, Taipei Medical University, Taipei, Taiwan.

250 Wuxing Street, Taipei 11031, Taiwan;

E-mail: [chaurjongh@tmu.edu.tw](mailto:chaurjongh@tmu.edu.tw)

**ROI coordinates used in this study (Montreal Neurological Institute system, radius: 6mm)**

|  | X | Y | Z |
| --- | --- | --- | --- |
| M1 (L) | -38 | -22 | 56 |
| SMA (L) | -5 | -4 | 57 |
| PCG (L) | -30 | -30 | 60 |
| PMd (L) | -22 | -13 | 57 |
| PMv (L) | -49 | -1 | 38 |
| M1 (R) | 38 | -22 | 56 |
| SMA (R) | 5 | -4 | 57 |
| PCG (R) | 37 | -34 | 53 |
| PMd (R) | 28 | -10 | 54 |
| PMv (R) | 53 | 0 | 25 |

|  | X | Y | Z |
| --- | --- | --- | --- |
| PCC (L) | -8 | -56 | 26 |
| amPFC (L) | -6 | 52 | -2 |
| IPL (L) | -44 | -74 | 32 |
| Rsp (L) | -14 | -52 | 8 |
| AnHip (L) | -28 | -12 | -20 |
| PCC (R) | 8 | -56 | 26 |
| amPFC (R) | 6 | 52 | -2 |
| IPL (R) | 44 | -74 | 32 |
| Rsp (R) | 14 | -52 | 8 |
| AnHip (R) | 28 | -12 | -20 |
